# Lightweight Helmet Detection Algorithm Using an Improved YOLOv4 †

**DOI:** 10.3390/s23031256

**Published:** 2023-01-21

**Authors:** Junhua Chen, Sihao Deng, Ping Wang, Xueda Huang, Yanfei Liu

**Affiliations:** 1 School of Computer Science and Technology, Chongqing University of Posts and Telecommunications, Chongqing 400065, China; 2 Key Laboratory of Industrial Internet of Things & Networked Control, Chongqing University of Posts and Telecommunications, Chongqing 400065, China; 3 School of Artificial Intelligence, Chongqing University of Technology, Chongqing 400054, China

**Keywords:** helmet detection, YOLOv4, PP-LCNet, attention mechanism, feature fusion, SIoU

## Abstract

Safety helmet wearing plays a major role in protecting the safety of workers in industry and construction, so a real-time helmet wearing detection technology is very necessary. This paper proposes an improved YOLOv4 algorithm to achieve real-time and efficient safety helmet wearing detection. The improved YOLOv4 algorithm adopts a lightweight network PP-LCNet as the backbone network and uses deepwise separable convolution to decrease the model parameters. Besides, the coordinate attention mechanism module is embedded in the three output feature layers of the backbone network to enhance the feature information, and an improved feature fusion structure is designed to fuse the target information. In terms of the loss function, we use a new SIoU loss function that fuses directional information to increase detection precision. The experimental findings demonstrate that the improved YOLOv4 algorithm achieves an accuracy of 92.98%, a model size of 41.88 M, and a detection speed of 43.23 pictures/s. Compared with the original YOLOv4, the accuracy increases by 0.52%, the model size decreases by about 83%, and the detection speed increases by 88%. Compared with other existing methods, it performs better in terms of precision and speed.

## 1. Introduction

Safety management on building sites has steadily drawn more attention with the progress of industrialization. Personal protective equipment (PPE) plays a vital part in ensuring the personal safety of workers [1]. As a basic personal protective equipment, the safety helmet can reduce the impact on the human head to a certain extent and protect the human life safety when an accident occurs. However, accidents arising from workers not wearing safety helmets can be seen everywhere due to a lack of a particular sense of safety protection. Therefore, monitoring whether workers are wearing helmets is crucial to their safety. Traditional helmet inspection mainly consists of monitoring in the surveillance room and manual patrol at the construction site. The former requires inspectors to stare at the screen for long periods, which can cause eye fatigue and lead to misjudgments and missed inspections, while the latter requires a lot of time and labor. Motivated by this, new methods for detecting the wearing of safety helmets by construction site workers are rapidly emerging with the help of sensors and image analysis techniques [2].

Sensor-based detection is mostly carried out by mounting several sensors on the helmet and determining whether the helmet is being worn or not based on the information gathered. Kelm et al. [3] designed an automatic entrance. By embedding RFID and tags in the PPE, the automatic entrance can effectively detect the PPE wearing status of workers as they pass through this portal. However, it can only be detected when workers pass through, and the status after getting into the construction site is uncertain. Kim et al. [4] developed a system that connects a three-axis acceleration sensor to a helmet, by which it is possible not only to identify whether the helmet is worn or not, but also to detect whether the helmet is worn correctly. Zhang et al. [5] monitored the wearing condition by installing an infrared beam detector and a thermal infrared sensor inside the helmet. However, all of these methods increase the expense of detection, and sensors embedded in helmets or other locations can lead to concerns about privacy and health.

In the last few years, due to the rapid advancement of computer technology, it has become possible to apply GPUs for massively parallel computing to train large deep neural networks [6,7]. Object detection based on deep learning has received more and more attention as a non-invasive method. There are two types of existing object detection algorithms: one-stage algorithms and two-stage algorithms. The two-stage algorithms mainly consist of two steps. The first step is to generate a series of region proposals that contain information about the rough location of objects. The second step is to classify and locate the generated region proposals to obtain the detection result [8]. The two-stage algorithm is characterized by high accuracy. However, due to the complex model and many calculation parameters, the speed of the two-stage algorithms cannot reach the real-time monitoring requirements. The classic two-stage algorithms include R-CNN [9], fast R-CNN [10], and faster R-CNN [11]. Different from the two-stage algorithms, the one-stage algorithms do not have the step of generating region proposals, but directly regress the position and classification probability of the boundary box, enabling an increase in speed. Therefore, the one-stage algorithms are more applicable to real-time target detection. The one-stage algorithms include the YOLO series [12,13,14,15], CenterNet [16], and SSD [17].

The advancement of object detection has inspired the safety helmet detection method based on deep learning, and many investigators believe that the deep learning technology is an important way to address construction security management problems [18]. Fang et al. [19] developed a smart non-safety helmet detector on the basis of Faster R-CNN with an accuracy of more than 90% in various scenes, but it takes about 0.2 s to detect an image, which cannot achieve the real-time demand. Gu et al. [20] used multiscale training based on Faster R-CNN and added an anchor strategy to improve it, which eventually led to a 7% improvement in helmet detection accuracy. Due to the shortcomings of two-stage algorithms that cannot meet real-time, one-stage algorithms are increasingly favored by researchers. Shen et al. [21] presented a modified SSD safety helmet detection algorithm, which first uses the SSD network to obtain the rough location of the safety helmet and then compares it with the detection results of adjoining frames to increase the detection precision of small objects, but it is considerably slower. In a study by Wu et al. [22], a densely connected convolutional network [23] was used to substitute the backbone network of YOLOv3 [14], achieving a better detection performance with the same detection time.

However, in some current helmet detection algorithms, the algorithm based on two-stage has a large number of parameters and slow detection speed, making it hard to satisfy the real-time demands. Although the algorithm based on the one-stage has a higher speed, its accuracy is lower compared to the two-stage algorithm, and it performs poorly when trying to identify small and intensive objects. To address the problems mentioned above and make high accuracy and fast detection speed, this paper selects YOLOv4 [15] as the base network. First, to solve the issues of an excessive number of network parameters and slow detection speed, the improved YOLOv4 discarded the original CSPDarknet53 structure having a large number of parameters and turned to the lightweight network PP-LCNet [24] as the backbone network, which is a high-performance network focused on mobile devices proposed by the Baidu team in 2021, and is significantly superior to other lightweight networks such as ShuffleNetV2 [25], MobileNetV2 [26], MobileNetV3 [27], and GhostNet [28] in terms of inference latency and accuracy balance. Furthermore, since a large number of 3 × 3 convolutions in the network also generate a large number of parameters, deepwise separable convolution with high computational efficiency was introduced and the 3 × 3 convolutions present in all parts of YOLOv4 except the backbone network were replaced with depthwise separable convolutions, further reducing the number of parameters in the network. With the above lightweight improvements, the number of parameters in YOLOv4 can be significantly reduced. However, the improvement of lightweightedness will bring a decrease in precision and poor detection of small targets. Therefore, in order to reduce the impact of lightweight improvements without increasing the number of parameters, the following three methods are adopted in this paper. First, the coordinate attention mechanism was introduced and added to the three outputs of the backbone network PP-LCNet to enable the network to acquire inter-channel information and direction-related location information, which can help to locate the target better. Second, in order to fully integrate the high-level and low-level features of the image to distinguish the foreground from the background, an efficient feature structure for enhanced feature extraction, PANet and BiFPN (PB) module, was designed by combining the weighted bi-directional feature pyramid network BiFPN [29] and PANet [30]. Finally, a newly proposed SIoU [31] loss function was adopted as the loss function of the original YOLOv4 to settle the matter of not taking into account the mismatched orientation between the ground-truth and predicted boxes. The primary components of this paper include:

(1) To reduce the parametric number of the model and improve the detection speed of the model deployed in the devices, PP-LCNet was used as the backbone of YOLOv4 for extracting features, and the depthwise separable convolution was used to substitute the normal convolution in the neck and head parts.
(2) The lightweight coordinate attention mechanism module was applied and inserted behind the output of the backbone network, allowing the model to be more sensitive to object location and increasing recognition precision.
(3) To merge the semantic information and specific features related to small objects while minimally increasing parameters, an efficient feature fusion structure PB block was designed to integrate the different levels of features.
(4) The SIoU loss function was adopted as a replacement for the original CIoU [32] function to take full account of the effects of the distance, aspect ratio, and angle between the ground-truth and the prediction boxes, which can speed up the convergence of the model.

The remaining sections of this paper are organized as follows. In Section 2, the YOLOv4 algorithm will be introduced. In Section 3, the improved YOLOv4 algorithm proposed in this paper will be described in detail. In Section 4, some experimental findings are demonstrated and analyzed. Finally, in Section 5, we summarise this paper and give an outlook toward future research.

## 2. Background

The YOLOv4 algorithm is an end-to-end real-time target detection algorithm that provides significant improvements in both precision and speed compared to YOLOv3. Therefore, we propose an improved YOLOv4 to obtain a lightweight model and then facilitate the real-time detection of safety helmets.

The specific network structure of YOLOv4 consists of three parts: the backbone network CSPDarkNet53 (backbone) for extracting image features, the enhanced feature extraction network (neck) including SPP (spatial pyramid pooling) [33] structure and PANet structure for further feature extraction, and the predictive decoding part YOLOHead (head). CSPDarkNet53 is improved on the basis of DarkNet53 [14]. It firstly uses the CSPNet [34] structure and divides DarkNet53 into two sections, one of which maintains the original stacking, and the other is connected to the end directly after a slight processing phase. Secondly, to obtain better accuracy and generalization, the LeakyReLU [15] activation function from DarkNet53 is changed to a smoother Mish [35] activation function.

In the SPP structure, pooling kernels of different sizes (such as 1 × 1, 5 × 5, 9 × 9, 13 × 13) are used for maximum pooling, which allows for a more efficient increase in the received field and a significant separation of important contextual features.

The PANet module builds a feature pyramid, which first propagates high-level semantic features to the underlying network through two upsampling modules. After each upsampling process, it fuses the features with high-resolution information, which makes it better at detecting small targets. Then, the feature fusion is further enhanced using two downsampling modules to fully extract important features.

The head part is still adopting the detection head of YOLOv3 and uses multiple convolutions for the prediction of the extracted features. After training and testing the model, the loss function can be determined by a comparison of the obtained prediction results with the real labeled information.

## 3. Proposed Methodology

**Figure 1:**
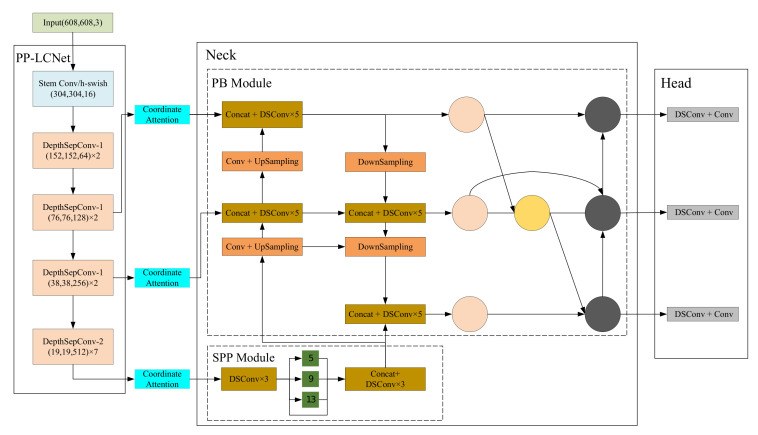
The architecture of the improved YOLOv4. DSConv represents the deepwise separable convolution, which is used in our method to replace the general convolution to decrease the network complexity.

Figure 1 shows the overall architecture of the improved YOLOv4. As shown in Figure 1, the improved YOLOv4 network structure proposed in this paper consists of four components: the backbone network PP-LCNet for feature extraction, the coordinate attention part for obtaining inter-channel relationships and location information, the neck part for information fusion including the SPP module and the PB module, and the head part for predictive decoding. Assuming that the size of the input image is (608, 608, 3), three feature maps of (76, 76, 128), (38, 38, 256), and (19, 19, 512) are obtained after extracting useful information about the target using the backbone network PP-LCNet. The three obtained feature maps are passed through the coordinate attention section to capture the channel and position information. Then, the feature information is integrated using the SPP and PB modules in the neck section. Finally, the feature maps are parsed in the head part to obtain the detection results.

### 3.1. Backbone: PP-LCNet

Due to the large number of layers in the backbone network CSPDarknet53 of YOLOv4, it can effectively extract deep-level feature information of images, which makes its object detection performance very excellent. However, this also leads to a more complex network structure, resulting in an excessive number of parameters and an increase in calculation time. Therefore, it is essential to reduce the quantity of parameters of the model so that it is capable of running on different edge devices in real time. In this paper, PP-LCNet is chosen as the backbone network for YOLOv4.

PP-LCNet uses DeapthSepCov proposed by MobileNetV1 [36] as the base module and applies several optimizations based on it. First, DepthSepCov without branches is used in PP-LCNet to increase the inference speed. Second, PP-LCNet replaces the ReLU [37] activation function of the backbone network with a better-performance H-Swish [27], which avoids a huge number of exponential operations. Third, the SE [38] module is inserted in the end layers of the network to expand the useful feature information. Finally, 5 × 5 convolutional kernels are used instead of 3 × 3 convolution in the deep layer to obtain a larger perceptual field.

**Figure 2:**
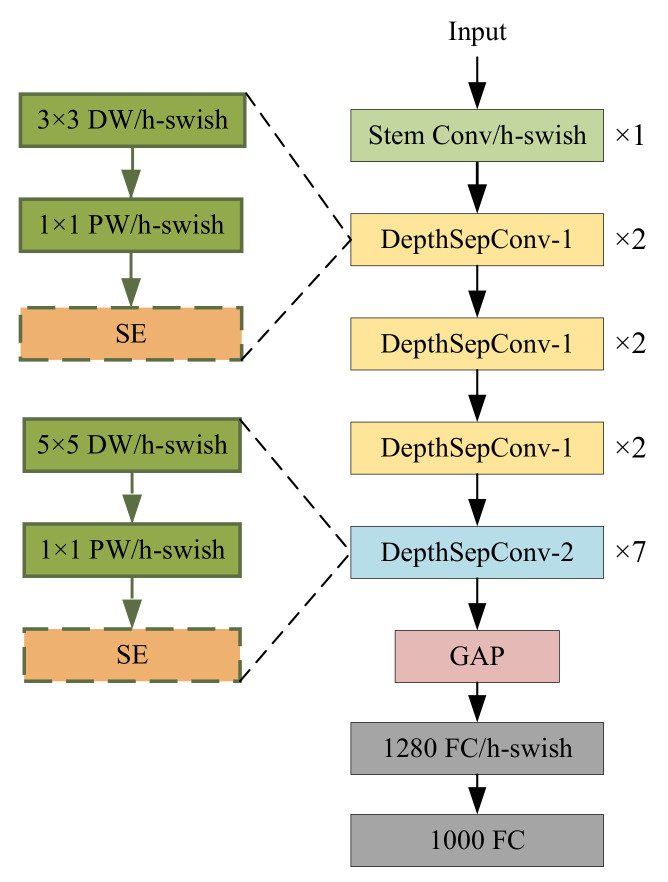
The structure of PP-LCNet. DW denotes the deepwsie convolution, PW denotes the point convolution, Stem denotes the standard convolution, GAP denotes the global average pooling, and FC denotes the fully-connected layer.

The network structure of PP-LCNet is shown in Figure 2. In order to make the PP-LCNet be the backbone network for YOLOv4, we remove the GAP and FC layers from the last three layers of the PP-LCNet and only use its first five layers for feature extraction. The output of the third, the fourth, and the fifth layer is taken as the input for the subsequent part.

### 3.2. Attention Mechanism: Coordinate Attention

In the actual environment, the effective features used for recognition in the images captured by the video only account for a minor portion, and the other features are more complex context information, which will generate a large amount of irrelevant information in the convolution computation. This irrelevant information will lead to some object details getting masked and increase the detection difficulty. To overcome the interference brought by the environment to the detection, this paper uses the attention mechanism to improve the detection accuracy. The attention mechanism can allocate finite computer resources to the more critical parts of the image and decrease the effect of other unrelated backgrounds and help our model to obtain more useful information. To balance the accuracy and the complexity, the coordinate attention mechanism [39] is adopted in this paper to significantly improve the performance of our model with extremely few additional parameters.

The coordinate attention module integrates the position details into the channel so that the region of interest gets more attention and the model can capture information in a larger area, which will effectively separate the target region from the background. That is to say, the coordinate attention mechanism allows the model to locate and distinguish the target region more accurately.

**Figure 3:**
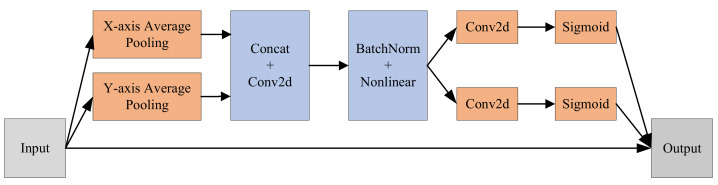
Coordinate attention structure.

The whole process of the coordinate attention mechanism can be generally described in three steps, as illustrated in Figure 3. The first step is coordinate information embedding. Specifically, the pooling operation is decomposed into two one-dimensional pooling operations along the *x*-axis and *y*-axis orientations to generate a couple of orientation-aware feature maps, so as to solve the difficulty of pooling operation for preserving position information. The second step is attention generation. The two feature maps generated in the first step are concatenated in the spatial dimension and channels are compressed using a convolution kernel of size 1 × 1. Next, the spatial information is encoded in the *x*-axis and *y*-axis directions using BatchNorm and Nonlinear. Then, the feature map is split into two independent tensors along the spatial dimension and the number of channels is adjusted to the same as the number of channels of the initial input feature map using a size of 1 × 1 convolution. Finally, the sigmoid activation function is applied to obtain the attention weights in two different directions. The last step is residual connection. The original input and the attention weights obtained in the second step are joined by the residuals to obtain the final result.

In this paper, the coordinate attention module is added following the output of PP-LCNet to provide the model with the ability to locate and recognize object regions more precisely.

### 3.3. PB Module

The low-level features of an image carry position details of the object, while the high-level features are rich in classification information. However, as the network deepens, the high-level features become more obvious, while the low-level features become more vague. Therefore, in order to make the feature map be characterized by more semantic features, the BiFPN structure is introduced. BiFPN can fully fuse various features, especially those of obscured or smaller objects in complex backgrounds, prevent the loss of low-level features, and effectively distinguish foreground from background. BiFPN is improved on the basis of the standard feature pyramid. Firstly, nodes with a single input edge are deleted, which means that the central nodes of the first and the last edges are removed. Then, all edges except the first and the last edges are added with a residual edge that connects the input to the output, merging more characteristics at a smaller cost. Finally, a base BiFPN module is formed, which can be repeated many times to achieve higher level feature fusion.

**Figure 4:**
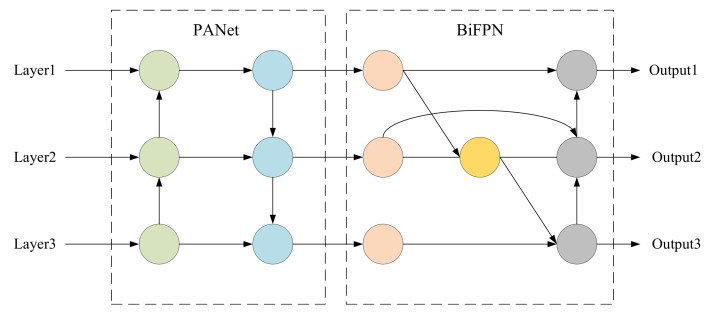
The structure of PB module.

In this paper, we combine PANet with BiFPN to construct an efficient feature fusion structure called PB module, which can enhance detection accuracy with a few parameters introduced. The structure of the PB module is shown in Figure 4. As shown in Figure 4, the PB module continues to use the original PANet to perform cross-scale weighted feature fusion and then imports the fused features into BiFPN for deeper information integration.

### 3.4. Depthwise Separable Convolution

The neck and the head parts in the original YOLOv4 and the proposed PB module contain a large number of 3 × 3 convolutional structures, which greatly increase network parameters and computation and then affect the detection speed. To decrease the complexity of the network, the depthwise separable convolution [40] is used to substitute the general 3 × 3 convolution in this paper to achieve an effective reduction of parameters.

**Figure 5:**
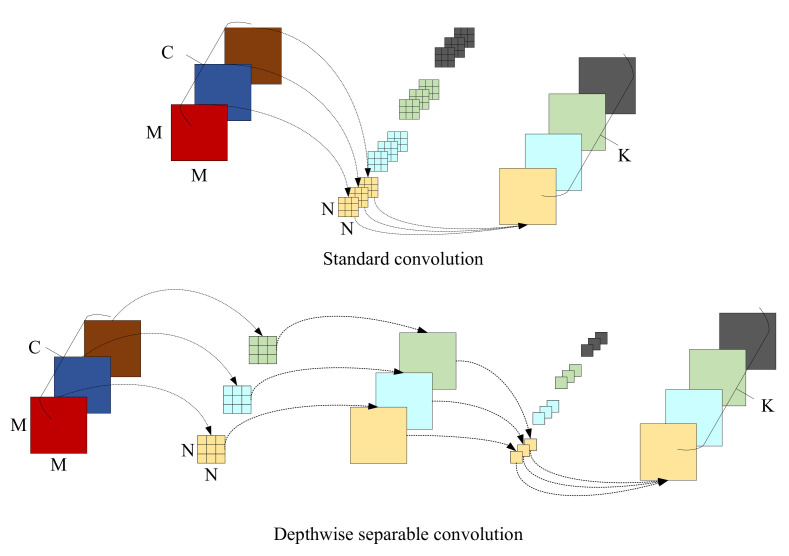
Comparison of general convolution and depthwise separable convolution.

Figure 5 shows the process of extracting features from the general convolution and the depthwise separable convolution, respectively. For images with three input channels, the standard convolution has only one step, with the same convolution process being performed on different input channels during each convolution. Unlike standard convolution, depthwise separable convolution is divided into two steps: depthwise convolution and point convolution. During the process of depthwise convolution, an individual filter is used to make a convolution operation for each channel of the input. During the point convolution, the dimensionality is increased with the use of a convolution kernel with the size of 1 × 1. Given an input size of M×M×C, a convolutional kernel size of N×N, and an output channel size of *K*, the proportion of the computation of depthwise separable convolution to that of general convolution is calculated as follows:

(1) $$\frac{M\times M\times C\times N\times N+K\times C\times M\times M}{M\times M\times C\times K\times N\times N}=\frac{1}{K}+\frac{1}{N^{2}}$$

In Equation (1), since the value of *N* is generally 3 and *K* is greater than 1, the depthwise separable convolution requires comparatively little computation.

### 3.5. Loss Function

As in the actual scenario, the detection of safety helmets may have features of large numbers, small targets, and intensive locations. In intensely distributed regions, given that the oriented disparity between the predicted box and the ground-truth box is not taken into account by CIoU, it might be the case that the predicted box has large leve ls of freedom and poor convergence rate of the match between the predicted box and the ground-truth box, which make the model suffer from mislocalization problems. Therefore, the SIoU loss function is introduced to replace the CIoU loss function in this paper.

The SIoU loss function is composed of four cost functions, which are angle cost, distance cost, shape cost, and IoU cost. First, the angle cost function Λ is given by the following equation:

(2) $$\Lambda =1-2\times \sin^{2}\left(\arcsin\left(\frac{c_{h}}{\sigma }\right)-\frac{\Pi }{4}\right),$$

where

$$c_{h}=\max\left(b_{c_{y}}^{gt},\;b_{c_{y}}^{pred}\right)-\min\left(b_{c_{y}}^{gt},\;b_{c_{y}}^{pred}\right),\;\sigma =\sqrt{{\left(b_{c_{x}}^{gt}-b_{c_{x}}^{pred}\right)}^{2}+{\left(b_{c_{y}}^{gt}-b_{c_{y}}^{pred}\right)}^{2}},$$

**Figure 6:**
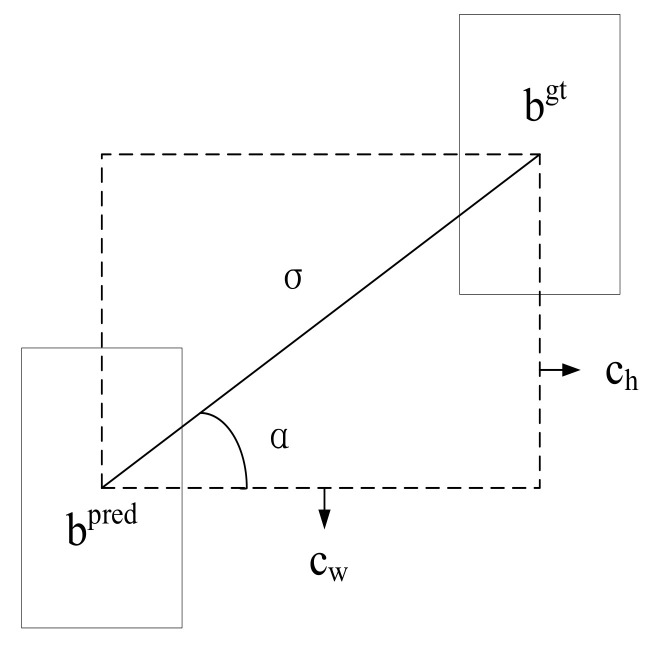
Diagram of SIoU loss function.

$b^{pred}$ and $b^{gt}$ denote the center point positions of the ground-truth box and predicted box, $\left(b_{c_{x}}^{gt},\;b_{c_{y}}^{gt}\right)$ and $\left(b_{c_{x}}^{pred},\;b_{c_{y}}^{pred}\right)$ are the coordinate positions of $b^{gt}$ and $b^{pred}$ respectively, as shown in Figure 6.

Considering the angle cost described above, the distance cost function Δ is redefined as:

(3) $$\Delta =\sum_{t=x,y}\left(1-e^{-\gamma \rho_{t}}\right),$$

where

$$\rho_{x}={\left(\frac{b_{c_{x}}^{gt}-b_{c_{x}}^{pred}}{c_{w}}\right)}^{2},\;\rho_{y}={\left(\frac{b_{c_{y}}^{gt}-b_{c_{y}}^{pred}}{c_{h}}\right)}^{2},\;\gamma =2-\Lambda .$$

The shape cost function is defined as:

(4) $$\Omega =\sum_{t=w,h}{\left(1-e^{-w_{t}}\right)}^{\theta },$$

where

$$\omega_{w}=\frac{\left|w-w^{gt}\right|}{\max\left(w,w^{gt}\right)},\;\omega_{h}=\frac{\left|h-h^{gt}\right|}{\max\left(h,h^{gt}\right)},$$

and (w,h) means the width and the height of the predicted box, $\left(w^{gt},h^{gt}\right)$ means the width, and the height of the ground-truth box; θ is applied to manipulate the extent of attention to the shape loss.

The IoU cost is defined as follows:

(5) $$L_{\mathrm{IoUCost}}=1-\mathrm{IoU}.$$

Therefore, the SIoU loss expression is described as:

(6) $$L_{\mathrm{SIoU}}=1-\mathrm{IoU}+\frac{\Delta +\Omega }{2}.$$

## 4. Experiment and Analysis

### 4.1. Dataset and Evaluation Criteria

The Safety Helmet Wearing Dataset (SHWD) [41] is used in this experiment. However, this dataset has problems such as incorrect labels and unlabeled labels, so we optimized it by verifying the annotation of the images and correcting the mislabeling. Finally, we obtain the optimized dataset containing 7004 images, with 7709 targets wearing helmets labeled as hat and 101,174 targets not wearing helmets labeled as person in all images.

The evaluation indicators used in this experiment are precision (P), recall (R), F1, average precision (mAP), frames per second (FPS), and model volume size. Precision is the correct rate of prediction in all samples with positive prediction. Recall is the correct rate of prediction in all truly positive samples. F1 is the harmonic average of the precision and the recall. The precision, recall, and F1 can be calculated from the following equations:

(7) $$P=\frac{TP}{TP+FP}$$

(8) $$R=\frac{TP}{TP+FN}$$

(9) $$F1=\frac{2PR}{P+R},$$

where TP means that the true category is positive and the detection result is positive, FP means that the true category is negative and the detection result is positive, and FN means that the true category is positive and the detection result is negative. The AP value of each category can be obtained by the area of the precision and recall curves (PR curves), and then the average of the two categories is taken to obtain the map value. AP and mAP are calculated as follows:

(10) AP=∫PRdr

(11) $$\;mAP=\frac{1}{k}\sum_{i=1}^{k}AP_{i},$$

where k is used to represent the number of categories; ${\mathrm{AP}}_{i}$ is used to represent the value of the i-th category.

### 4.2. Training Process and Results

The experiments of this paper are built under a 64-bit windows10 system. The processor is i7-10700H CPU, and the GPU is GeForce RTX 3060. The training environment is CUDA11.0, cuDNN 7.6.4, Python3.7, and PyTorch 1.7.

**Figure 7:**
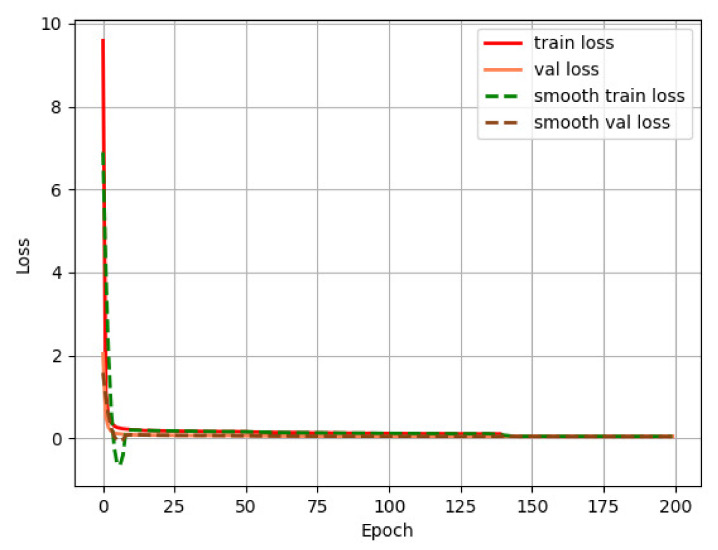
Loss value curve.

**Figure 8:**
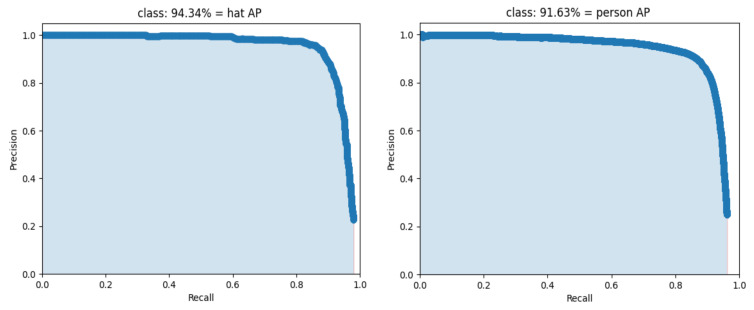
Precision–recall curve of the improved YOLOv4. The left image is the PR curve of hat and the right image is the PR curve of person.

In this paper, we use the concept of transfer learning to train the model with the weights of the pretrained backbone network. Transfer learning means transferring the model to a target domain with similar features after training with a large amount of data in a known domain. The use of the pretrained in advance allows the model to quickly acquire characteristic information in another new domain, decreases the training time to a certain degree, and speeds up the convergence of the network. First, the backbone part of the network is frozen, and its parameters are not involved in training, and the parameters of the other parts are trained to adjust the network to the new dataset. The epoch of the frozen part is set to 50, the batch size is set to 16, the initial learning rate is 0.001, the Adam optimizer is used, and the cosine annealing learning rate decay strategy is employed. After the training of the freezing phase, the backbone network is unfrozen, and all parameters of the network are involved in the training at this time. The epoch of the unfreezing phase is set to 150, and the batch size is set to 8. The final loss function curve and precision–recall curve are shown in Figure 7 and Figure 8, respectively.

### 4.3. Ablation Experiments

To demonstrate the effectiveness of the improved YOLOv4, an ablation experiment is adopted in this paper. First, the depthwise separable convolution is employed to displace the general 3 × 3 convolution located in the neck and head parts of YOLOv4. Second, the backbone network CSPDarkNet53 of YOLOv4 is substituted with PP-LCNet. Third, the coordinate attention module is applied and inserted into the output part of the backbone network PP-LCNet. After that, the proposed PB module is taken as an enhanced feature extraction network for YOLOv4. Finally, the loss function CIoU is modified to SIoU. Table 1 shows the ablation experimental results, where DSC denotes depthwise separable convolution, and CA denotes the coordinate attention module.

Model-1 denotes the original YOLOv4 model, and Model-2 denotes the model after applying the depthwise separable convolution in the neck and head parts of YOLOv4. The accuracy of Model-2 only decreases by 0.6% compared to Model-1, while the model size decreases by 44.14%. This demonstrates that the depthwise separable convolution can greatly lower the number of parameters with little impact on accuracy. Model-3 adopts PP-LCNet as a replacement for YOLOv4’s backbone network CSPDarknet53 on the basis of Model-2, which further reduces its model size to 38.75 M, equivalent to 15.89% of the original network, despite a 3.12% decrease in accuracy compared to the original network Model-1. Although the use of PP-LCNet can significantly simplify the complexity of the network, it is simultaneously coupled with a decrease in precision. To solve this problem, the coordinate attention module is inserted after the three outputs of the backbone network in Model-4, which provides a 0.75% improvement in accuracy compared to Model-3, but the model size is basically unchanged. The results illustrate that the coordinate attention mechanism is capable of increasing the accuracy of the network with almost no additional cost. Model-5 uses the PB module presented in this paper as a replacement for PANet structure of the original network based on Model-4. Compared with Model-4, the accuracy increases by 1.2%, and the model size increases slightly by 2.89M. Finally, Model-6 employs the SIoU loss function instead of the CIoU loss function. Compared with Model-1, the accuracy increases by 0.52%, and the model size is only 17.16% of the original network. The experiments show that the optimized YOLOv4 algorithm provides good detection precision and a small model size.

**Figure 9:**
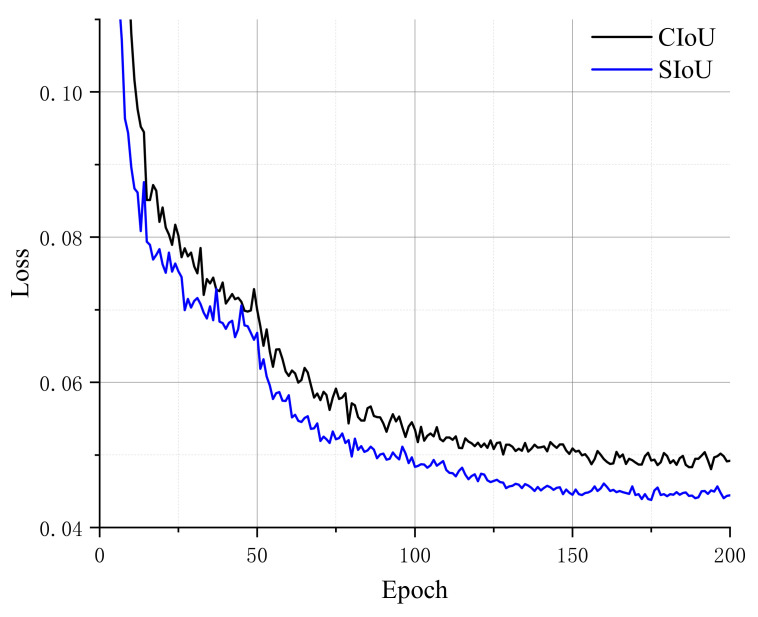
Comparison of CIoU and SIoU in training.

Furthermore, to validate the performance of the SIoU loss function during the training phase, we utilize SIoU and CIoU on the same dataset with the same parameters and compare the loss variation curves throughout the training phase, as shown in Figure 9.

From the loss curves of CIoU and SIoU, it can be seen that the loss values after using the SIoU loss function are generally lower than that after using the CIoU loss function, and the convergence of SIoU is faster, which proves that the model has better performance in inference after using the SIoU loss function.

### 4.4. Comparative Experiments

**Figure 10:**
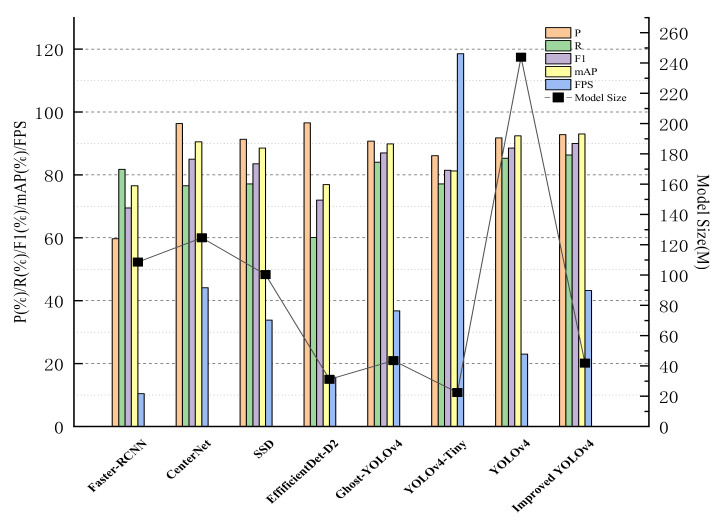
Comparison of histograms of the test results of different algorithms.

To further evaluate the validity of the algorithm in this paper, we compare the improved YOLOv4 with other advanced algorithms under the same experimental environment with the same evaluation criteria, as shown in Table 2. In order to visualize the comparison result, a histogram is plotted on the basis of the data from the experiment, as presented in Figure 10. From the data in Table 2, it can be concluded that the YOLOv4 algorithm can achieve high accuracy detection of safety helmet wearing with the advantage of its construction, and the mAP can reach 92.46%, but its detection speed is only 23.02 pictures/s. That is to say, it takes 43 ms to detect an image, and the model size is up to 243.92 M, which is not desirable to be deployed to the embedded devices for real-time detection. The improved YOLOv4 algorithm introduced in this paper can increase the mAP to 92.98% while reducing the model size by 83% and improving the detection speed to 43.24 picture/s, which means it only takes 23 ms to detect an image, almost 1.87 times the FPS of YOLOv4. Although the FPS of the improved YOLOv4 is not as good as that of YOLOv4-Tiny [42] and its model size is larger than that of YOLOv4-Tiny, its P, R, F1, and mAP are 6.66%, 9.25%, 8.5%, and 11.72% higher than those of YOLOv4-Tiny, respectively. Compared with Ghost-YOLOv4 [43], which replaces the YOLOv4 backbone with GhostNet, the improved YOLOv4 has 2.02%, 2.28%, 3.15%, and 6.64 pictures/s increments in the P, R, F1, mAP, and FPS, respectively, and a 1.72M decrement in model size. For faster R-CNN, CenterNet, SSD, and EffificientDet-D2 [29], the improved YOLOv4 is superior to them with regard to detection accuracy, speed, and model size.

**Figure 11:**
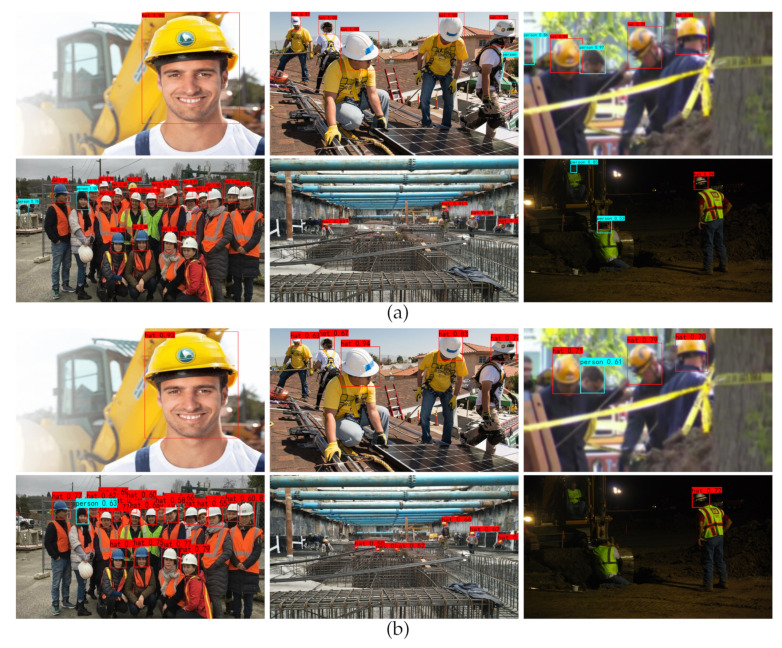

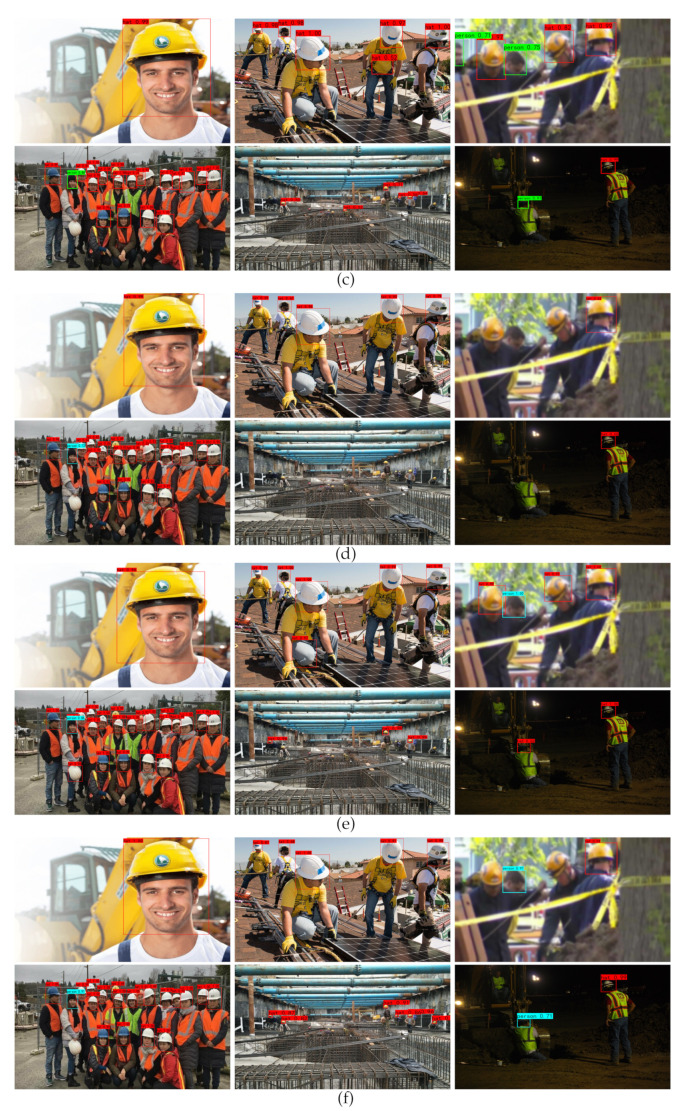

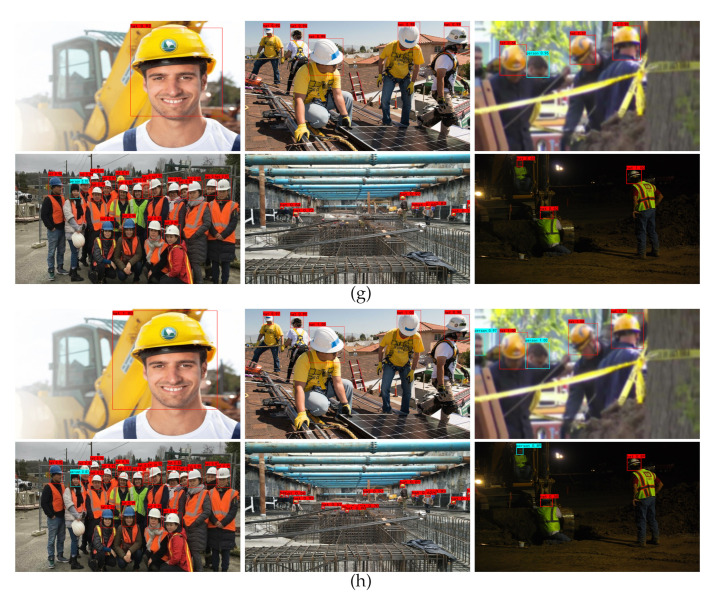
Comparison results of actual detection with different algorithms: (**a**) Faster R-CNN; (**b**) CenterNet; (**c**) SSD; (**d**) EfficientDet-D2; (**e**) Ghost-YOLOv4; (**f**) YOLOv4-Tiny; (**g**) YOLOv4; and (**h**) Improved YOLOv4. The images in the first row of each algorithm show the detection results for a single target, a small number of targets and blurred targets, respectively, while the images in the second row of each algorithm show the detection results for densely obscured targets, small targets at a long distance and poorly light targets, respectively.

To validate the reliability of the improved algorithm in some complex scenarios, we selected images from different environments for safety helmet detection. The results are shown on Figure 11. We can conclude the following observations from Figure 11.

For a single target, all models have higher accuracy, among which faster-RCNN, YOLOv4-Tiny, and the improved YOLOv4 have the highest accuracy.

For a small number of targets, faster R-CNN, SSD, and Ghost-YOLOv4 all have false detections. The accuracy of other models is not as high as that of the improved YOLOv4.

For the blurred targets, only faster R-CNN, SSD, and the improved YOLOv4 can detect the targets completely, and the improved YOLOv4 has the best effect, while other models have some missing detections. For example, CenterNet, Ghsot-YOLOv4, and YOLOv4 have one missing detection, YOLOv4-Tiny has three missing detections, and EfficientDet-D2 has four missing detections.

For the densely occluded targets, the improved YOLOv4 can correctly identify all targets, including some severely occluded targets, while other models have certainly missed detections when identifying severely occluded targets. Some models also have false detection. For example, faster R-CNN identifies the car tires in the test image as the category of person, and Ghost-YOLOv4 recognizes a helmet that is not worn on the head as the category of hat.

For the small targets at long range, all models have missed detection, but the improved YOLOv4 has a low missed detection rate, with only two targets missed in the current test image, while YOLOv4 misses eight targets, and all other models have more than 10 missed detections. Therefore, for lightweight networks, the improved YOLOv4 algorithm achieves better effectiveness in the detection of small targets.

For the dim and poorly light targets, the improved YOLOv4 model shows a good test result, while other models have false and missed detections, such as CenterNet, SSD, EfficientDet-D2, Ghost-YOLOv4, and YOLOv4-Tiny. In addition, faster R-CNN, SSD, and YOLOv4-Tiny identify a worker wearing a helmet and working with head down as the category of person, and YOLOv4 identifies a worker driving without a helmet as the category of hat. The above detection findings demonstrate that the improved YOLOv4 algorithm dramatically increases the detection in all kinds of scenarios.

**Figure 12:**
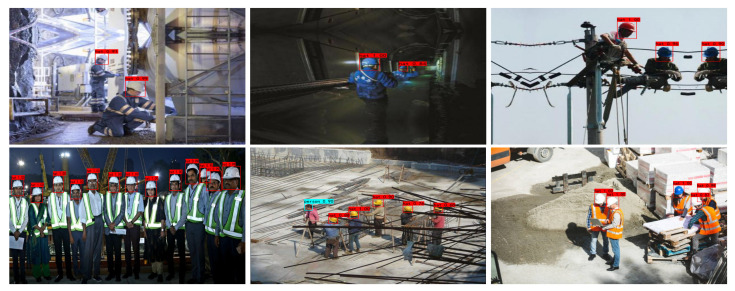
The three images in the first row are from Hard Hat Dataset and the three images in the second row are from Helmet Dataset.

Figure 12 represents the test results using the improved YOLOv4 proposed in this paper on another two datasets, Hard Hat Dataset [44] and Helmet Dataset [45]. The experimental results further demonstrate the effectiveness of the improved YOLOv4 for helmet wearing detection, which verifies the proposed model have well generalization ability.

## 5. Conclusions

To decrease the accidents resulting from workers not wearing safety helmets in construction sites, this paper presents a lightweight safety helmet wearing detection algorithm. On the basis of the YOLOv4 algorithm, PP-LCNet is adopted to replace CSPDarknet53 as the backbone network, and the depthwise separable convolution is used as a substitute for the normal convolution in the network structure, significantly squeezing the model volume. Furthermore, to compensate for the decreased accuracy after the modification, the coordinate attention module is added to the output position of the backbone network, which can effectively distinguish the foreground and background while not expanding the model space, and then improve the detection accuracy. After that, the PB module is constructed by integrating PANet and BiFPN to reinforce feature fusion and acquire effective features. Finally, the SIoU loss function is applied to significantly minimize the cost in degrees of freedom. The experimental results demonstrate that, compared with the original YOLOv4 model, the accuracy of the improved YOLOv4 model is increased by 0.52%, and its model size is reduced by about 83%. Compared with other networks, the improved YOLOv4 also provides a better combination of accuracy and speed for safety helmet detection tasks. Although the improved YOLOv4 proposed in this paper can greatly reduce the model size and increase the detection speed while maintaining a high level of accuracy, there are still problems that can be optimized. For example, in the comparative experiments, although the improved YOLOv4 has achieved better results compared with other models for small targets at a long distance, there are still cases of missing detection due to too small targets. In the future, we will further investigate the detection problem for the special case of small targets.

## Figures and Tables

**Table 1:** Results of ablation experiments.

Model	DSC	PP-LCNet	CA	PB	SIoU	AP		mAP	ModelSize
						Hat	Person		
Model-1	×	×	×	×	×	93.15%	91.77%	92.46%	243.92M
Model-2	*√*	×	×	×	×	92.71%	91.00%	91.86%	136.13M
Model-3	*√*	*√*	×	×	×	88.85%	89.38%	89.34%	38.75M
Model-4	*√*	*√*	*√*	×	×	89.36%	92.82%	90.09%	38.99M
Model-5	*√*	*√*	*√*	*√*	×	90.54%	92.03%	91.29%	41.88M
Model-6	*√*	*√*	*√*	*√*	*√*	94.34%	91.63%	92.98%	41.88M

**Table 2:** Comparison results of different models.

Model	Precision (%)	Recall (%)	F1 (%)	mAP (%)	FPS	Model Size (M)
Faster R-CNN [11]	59.74	81.78	69.50	76.53	10.45	108.64
CenterNet [16]	96.34	76.56	85.00	90.54	44.14	124.61
SSD [17]	91.31	77.11	83.50	88.53	33.86	100.27
EffificientDet-D2 [29]	96.51	60.10	72.00	76.92	15.64	31.2
Ghost-YOLOv4 [43]	90.77	84.07	87.00	89.83	36.78	43.6
YOLOv4-Tiny [42]	86.12	77.10	81.50	81.26	118.52	22.42
YOLOv4 [15]	91.80	85.30	88.50	92.46	23.02	243.92
Improved YOLOv4	92.78	86.35	90.00	92.98	43.24	41.88

## Data Availability

The SHWD dataset provided in this study is available at https://github.com/njvisionpower/Safety-Helmet-Wearing-Dataset, accessed on 20 December 2022.

## References

[1] Wang Z., Wu Y., Yang L., Thirunavukarasu A., Evison C., Zhao Y. (2021). Fast personal protective equipment detection for real construction sites using deep learning approaches. Sensors.

[2] Han K., Zeng X. (2021). Deep learning-based workers safety helmet wearing detection on construction sites using multi-scale features. IEEE Access.

[3] Kelm A., Laußat L., Meins-Becker A., Platz D., Khazaee M.J., Costin A.M., Helmus M., Teizer J. (2013). Mobile passive Radio Frequency Identification (RFID) portal for automated and rapid control of Personal Protective Equipment (PPE) on construction sites. Autom. Constr..

[4] Kim S.H., Wang C., Min S.D., Lee S.H. (2018). Safety helmet wearing management system for construction workers using three-axis accelerometer sensor. Appl. Sci..

[5] Zhang H., Yan X., Li H., Jin R., Fu H. (2019). Real-time alarming, monitoring, and locating for non-hard-hat use in construction. J. Constr. Eng. Manag..

[6] Han K., Yang Q., Huang Z. (2020). A two-stage fall recognition algorithm based on human posture features. Sensors.

[7] Han K., Peng J., Yang Q., Tian W. (2021). An end-to-end dehazing Siamese region proposal network for high robustness object tracking. IEEE Access.

[8] Zha M., Qian W., Yi W., Hua J. (2021). A lightweight YOLOv4-Based forestry pest detection method using coordinate attention and feature fusion. Entropy.

[9] Girshick R., Donahue J., Darrell T., Malik J. Rich feature hierarchies for accurate object detection and semantic segmentation. Proceedings of the IEEE Conference on Computer Vision and Pattern Recognition.

[10] Girshick R. Fast r-cnn. Proceedings of the IEEE international Conference on Computer Vision.

[11] Ren S., He K., Girshick R., Sun J. Faster r-cnn: Towards real-time object detection with region proposal networks. Proceedings of the 28th International Conference on Neural Information Processing Systems.

[12] Redmon J., Divvala S., Girshick R., Farhadi A. You only look once: Unified, real-time object detection. Proceedings of the IEEE Conference on Computer Vision and Pattern Recognition.

[13] Redmon J., Farhadi A. YOLO9000: Better, faster, stronger. Proceedings of the IEEE Conference on Computer Vision and Pattern Recognition.

[14] Redmon J., Farhadi A. (2018). Yolov3: An incremental improvement. arXiv.

[15] Bochkovskiy A., Wang C.Y., Liao H.Y.M. (2020). Yolov4: Optimal speed and accuracy of object detection. arXiv.

[16] Zhou X., Wang D., Krähenbühl P. (2019). Objects as points. arXiv.

[17] Liu W., Anguelov D., Erhan D., Szegedy C., Reed S., Fu C.Y., Berg A.C. (2016). Ssd: Single shot multibox detector. Proceedings of the European Conference on Computer Vision.

[18] Park C., Lee D., Khan N. (2020). An analysis on safety risk judgment patterns towards computer vision based construction safety management. Proceedings of the Creative Construction e-Conference 2020.

[19] Fang Q., Li H., Luo X., Ding L., Luo H., Rose T.M., An W. (2018). Detecting non-hardhat-use by a deep learning method from far-field surveillance videos. Autom. Constr..

[20] Gu Y., Xu S., Wang Y., Shi L. An advanced deep learning approach for safety helmet wearing detection. Proceedings of the 2019 International Conference on Internet of Things (iThings) and IEEE Green Computing and Communications (GreenCom) and IEEE Cyber, Physical and Social Computing (CPSCom) and IEEE Smart Data (SmartData).

[21] Shen J., Xiong X., Li Y., He W., Li P., Zheng X. (2021). Detecting safety helmet wearing on construction sites with bounding-box regression and deep transfer learning. Comput.-Aided Civ. Infrastruct. Eng..

[22] Wu F., Jin G., Gao M., Zhiwei H., Yang Y. Helmet detection based on improved YOLO V3 deep model. Proceedings of the 2019 IEEE 16th International Conference on Networking, Sensing and Control (ICNSC).

[23] Huang G., Liu Z., Van Der Maaten L., Weinberger K.Q. Densely connected convolutional networks. Proceedings of the IEEE Conference on Computer Vision and Pattern Recognition.

[24] Cui C., Gao T., Wei S., Du Y., Guo R., Dong S., Lu B., Zhou Y., Lv X., Liu Q. (2021). PP-LCNet: A Lightweight CPU Convolutional Neural Network. arXiv.

[25] Ma N., Zhang X., Zheng H.T., Sun J. Shufflenet v2: Practical guidelines for efficient cnn architecture design. Proceedings of the European Conference on Computer Vision (ECCV).

[26] Sandler M., Howard A., Zhu M., Zhmoginov A., Chen L.C. Mobilenetv2: Inverted residuals and linear bottlenecks. Proceedings of the IEEE Conference on Computer Vision and pattern Recognition.

[27] Howard A., Sandler M., Chu G., Chen L.C., Chen B., Tan M., Wang W., Zhu Y., Pang R., Vasudevan V. Searching for mobilenetv3. Proceedings of the IEEE/CVF International Conference on Computer Vision.

[28] Han K., Wang Y., Tian Q., Guo J., Xu C., Xu C. Ghostnet: More features from cheap operations. Proceedings of the IEEE/CVF Conference on Computer Vision and Pattern Recognition.

[29] Tan M., Pang R., Le Q.V. Efficientdet: Scalable and efficient object detection. Proceedings of the IEEE/CVF Conference on Computer Vision and Pattern Recognition.

[30] Liu S., Qi L., Qin H., Shi J., Jia J. Path aggregation network for instance segmentation. Proceedings of the IEEE Conference on Computer Vision and Pattern Recognition.

[31] Gevorgyan Z. (2022). SIoU Loss: More Powerful Learning for Bounding Box Regression. arXiv.

[32] Zheng Z., Wang P., Liu W., Li J., Ye R., Ren D. Distance-IoU loss: Faster and better learning for bounding box regression. Proceedings of the AAAI Conference on Artificial Intelligence.

[33] He K., Zhang X., Ren S., Sun J. (2015). Spatial pyramid pooling in deep convolutional networks for visual recognition. IEEE Trans. Pattern Anal. Mach. Intell..

[34] Wang C.Y., Liao H.Y.M., Wu Y.H., Chen P.Y., Hsieh J.W., Yeh I.H. CSPNet: A new backbone that can enhance learning capability of CNN. Proceedings of the IEEE/CVF Conference on Computer Vision and Pattern Recognition Workshops.

[35] Misra D. (2019). Mish: A self regularized non-monotonic neural activation function. arXiv.

[36] Howard A.G., Zhu M., Chen B., Kalenichenko D., Wang W., Weyand T., Andreetto M., Adam H. (2017). Mobilenets: Efficient convolutional neural networks for mobile vision applications. arXiv.

[37] Agarap A.F. (2018). Deep learning using rectified linear units (relu). arXiv.

[38] Hu J., Shen L., Sun G. Squeeze-and-excitation networks. Proceedings of the IEEE Conference on Computer Vision and Pattern Recognition.

[39] Hou Q., Zhou D., Feng J. Coordinate attention for efficient mobile network design. Proceedings of the IEEE/CVF Conference on Computer Vision and Pattern Recognition.

[40] Chollet F. Xception: Deep learning with depthwise separable convolutions. Proceedings of the IEEE Conference on Computer Vision and Pattern Recognition.

[41] njvisionpower (2019). Safety-Helmet-Wearing-Dataset. https://github.com/njvisionpower/Safety-Helmet-Wearing-Dataset.

[42] Jiang Z., Zhao L., Li S., Jia Y. (2020). Real-time object detection method based on improved YOLOv4-tiny. arXiv.

[43] Chen J., Deng S., Huang X., Yang X., Yan D. Safety Helmet Wearing Detection Based on A Lightweight YOLOv4 Algorithm. Proceedings of the 2022 IEEE International Conferences on Internet of Things (iThings) and IEEE Green Computing & Communications (GreenCom) and IEEE Cyber, Physical & Social Computing (CPSCom) and IEEE Smart Data (SmartData) and IEEE Congress on Cybermatics (Cybermatics).

[44] (2020). Hard Hat Dataset. https://makeml.app/datasets/hard-hat-workers.

[45] Wu J., Cai N., Chen W., Wang H., Wang G. (2019). Automatic detection of hardhats worn by construction personnel: A deep learning approach and benchmark dataset. Autom. Constr..

